# TBX2 Identified as a Potential Predictor of Bone Metastasis in Lung Adenocarcinoma via Integrated Bioinformatics Analyses and Verification of Functional Assay

**DOI:** 10.7150/jca.31636

**Published:** 2020-01-01

**Authors:** Huajian Yu, Fangyu Zhao, Jing Li, Kechao Zhu, Hechun Lin, Zhen Pan, Miaoxin Zhu, Ming Yao, Mingxia Yan

**Affiliations:** 1 State Key Laboratory of Oncogenes and Related Genes, Shanghai Cancer Institute, Renji Hospital, Shanghai Jiao Tong University School of Medicine, Shanghai, China;; 2 Department of Orthopedics, Shanghai Jiao Tong University Affiliated Sixth People's Hospital, Shanghai, China.; 3 Fudan University Shanghai Cancer Center, Shanghai Medical College, Fudan University, Shanghai, China.

**Keywords:** Integrated bioinformatics analysis, TBX2, Bone metastasis, Lung adenocarcinoma.

## Abstract

**Objective:** Bone metastasis from patients with advanced lung adenocarcinoma (LAC) is a very serious complication. To better understand the molecular mechanism, our current study sheds light on identification of hub genes mediating bone metastatic spread by combining bioinformatic analysis with functional verification.

**Methods:** First, we downloaded a lung adenocarcinoma dataset (GSE76194) from Gene Expression Omnibus, analyzed differentially expressed genes (DEGs) through Limma package in R software and constructed a protein-protein interaction network. Based on that preliminary data, we further performed modular and topological analysis using Cystoscope to obtain biological connected genes. Through literature searching and performing mRNA expression analysis on the other independent public dataset (GSE10799), we finally focused on TBX2. Functional effects of TBX2 were performed in tumorigenicity assays including migration and invasion assays, cell proliferation assay, and cell cycle assay. In addition, mechanically, we found enriched pathways related to bone metastasis using Gene Set Enrichment Analysis (GSEA) and validated our results by western blot.

**Result:** A total of 1132 significant genes were sorted initially. We selected common significant genes (log FC>2; p<0.01) from both the biological network data and microarray data. In total, 44 such genes were identified. we found TBX2, along with 10 other genes, to be reported with relevance to bone metastasis in other cancer types. Moreover, TBX2 showed significantly higher expression levels in patients that were found positive for metastasis to bone marrow compared to patients that did not exhibit this type of metastasis in the other separated cohort (GSE10799). Thus, we finally focused on TBX2. We found that TBX2 had detectable expression in LAC cell lines and silencing endogenous TBX2 expression in A549 and H1299 cell lines markedly suppressed migration and invasion, cell proliferation and arrested cell-cycle. Pathway enrichment analyses suggested that TBX2 drove LAC oncogenesis and metastasis through various pathways with epithelial mesenchymal transition (EMT) figuring prominently in the bone metastatic group, which was evidenced by western blot.

**Conclusion**: Collectively, TBX2 plays as a potential predictor of bone metastasis from LAC, yielding a better promise view towards “driver” gene responsible for bone metastasis.

## Introduction

Lung adenocarcinoma is the leading cause of cancer-related mortality among all cancers. Like other cancers, metastasis results in the highest lethality rates 1. Bone is the preferential site of metastasis in lung adenocarcinoma (LAC). Bone metastasis develops in approximately 30 to 40% of patients with advanced lung cancer 2. Many patients with lung cancer are in advanced stages of the disease at the time of diagnosis. The 5-year survival rate is lower than 20% and the mean survival after bone metastasis of lung cancer is 9.7 months 3. Patients with bone metastasis of advanced LAC are faced with an increased risk of bone fracture, unbearable bone pain, loss of functional independence on daily life, in addition to diminished overall survival outcomes 4. Once the sequence of events leading to the progression of tumor cell invasion and metastasis begins, bone metastasis will occur shortly 5. Effective molecular biomarkers conferring robust dissemination activity for early diagnosis and therapeutic options are urgently needed.

The Gene Expression Omnibus (GEO) database is a public functional genomics data repository providing the opportunity to explore, analyze, and visualize expression data through the method of the data mining in various cancers 6. Because of the limitation of large-scale functional screening method, many markers have already been proposed but the functional role rarely investigated. Bone metastasis is frequent with high rates of recurrence and mortality in LAC. In the present study, we found several high potential biomarkers for bone metastasis of LAC via integrating multiple bioinformatics approaches. Our present study uncovered the functional role of TBX2 in LAC and confirmed that knockdown of TBX2 inhibited cell migration and invasion, affected epithelial-mesenchymal transition (EMT), and also significantly suppressed cell growth through induction of cell-cycle arrest. Collectively, our innovative method and findings overcome the shortcomings of traditional analytical methods and will have suggestive effects on diagnosis and individualized treatment of advanced bone metastasis of lung cancer.

## Materials and methods

### Raw biological data

Gene expression data of the microarray GSE76194 was obtained from Gene Expression Omnibus and probes were switched into gene symbols based on the platform of GPL570 (Affymetrix Human Genome U133 Plus 2.0 Array). RNA from five pairs of parental and corresponding bone metastatic lung cancer cell lines of Chinese origin were collected.

### Data preprocessing and normalization

CEL files and the probe annotation files were downloaded and the gene expression profile was obtained, following robust multichip average background correction, quantile normalizing and calculation of expression using the affy package (Version 1.58.0) 7. Subsequently, a linear model was fitted and empirical Bayes statistics were computed by Limma package (Version 3.36.2) 8. Significant genes were sorted between groups of parental and bone metastatic cell lines. P<0.01 and |Log2(FC)|>0.7 were considered as initial standard of screening.

### Protein-protein interaction network (PPI) construction

To obtain functional links between proteins, we constructed a giant PPI network of the shortlisted genes by uploading preliminary screening results into The Search Tool for the Retrieval of Interacting Genes/Proteins (STRING; https://string-db.org/) database 9. Subsequently, the PPI network was reconstructed with Cytoscape software (Version 3.5.1). In current study, a combined MCS score of >0.4 (Medium confidence score) was considered to be significant.

### Module identification and enrichment analysis

A cluster search algorithm, the Molecular Complex Detection (MCODE) plugins were used to find highly interconnected regions clusters on the basis of vertex weighting by local neighborhood density and outward traversal from a locally dense seed protein, like protein complexes or parts of pathways in protein-protein interaction network 10. Modules with scores ≥5, the number of nodes >20, node score cutoff≥0.4, K-core≥2 and max depth = 100 were considered as significant 11.

### Identification of hub candidates from topological analysis

To explore important nodes in biological networks, we employed a topological analysis strategy by making good use of CytoHubba plugin of Cytoscape. It came into the limelight as it played significant role in the exploration of central elements of biological networks by measuring nodes by their network features. Scores were granted to each node in a preloaded PPI network with eleven scoring methods in one stop shopping way including Degree, Edge Percolated Component, Maximum Neighborhood Component, Density of Maximum Neighborhood Component, Maximal Clique Centrality and six centralities (Bottleneck, EcCentricity, Closeness, Radiality, Betweenness, and Stress based on shortest paths, of which MCC was plausible as its better performance on the precision of predicting essential proteins in the PPI network 12. In this study, we chose the nodes with MCC>=6 as clue to finally find significant hub candidates 12.

### Hub gene identification and functional enrichment analysis

Funrich Software (Version 3.1.3, http://funrich.org/index.html) was used to analyze the overlapping DEGs. A clustering analysis of up and down regulation genes was performed using the Pheatmap package in R statistical software. Functional enrichment analyses of Candidate DEGs including GO annotation analysis and Kyoto Encyclopedia of Genes and Genomes (KEGG) pathway enrichment analysis of DEGs were carried out using DAVID 13, 14. The genes were assigned to functional groups based on molecular function, biological processes and biological pathways. Gene counts≥3 was considered as the cut-off criterion. P-value <0.05 based upon hypergeometric test was set as significant and corrected by Benjamini and Hochberg FDR to test its significance.

### Validation of Clinical expression and survival analysis in silico

Expression data of patients with bone metastases from lung cancer (GSE10799) including 16 bone marrow samples from lung cancer metastasis patients, 9 of which were free of metastatic to bone marrow, 7 of which was positive of metastatic to bone marrow was also downloaded for further study 15.Moreover, the Kaplan-Meier Plotter database (http://kmplot.com/analysis/) was used to evaluate the prognostic values with background lung cancer database. Patients were stratified into two groups according to the median mRNA level. Survival outcome for first progression (FP), HRs and p-values were summarized and the log-rank tests were used to analyze differences in survival time.

### Gene Set Enrichment Analysis (GSEA) of mRNA profiling

Mounting evidence have shown that Gene Set Enrichment Analysis (GSEA) (Version 3.0) is play a pivot role in yielding additional insights into the common biologic pathways involved in various cancer pathogenesis 16. Here, classified as bone metastasis and parental group, samples were analyzed for its biologic pathway by GSEA. The GO gene sets biological process database (c5.bp.v4.0) from the Molecular Signatures Database (MsigDB). The value of the False Discovery Rate (FDR) <0.25 was considered to be well-established cutoff to determine enrichment terms. The NES (Normalized Enrichment Score) was utilized to compare the analysis results across gene sets.

### Cell lines and cell culture

Six human lung adenocarcinoma cell lines NCI-H1299、A549、NCI-H1975、PC-14、PC-9 were purchased from the American Type Culture Collection and were cultured using Dulbecco's modification of Eagle medium containing high glucose, supplemented with 10% fetal bovine serum (Biowest, South America Origin) with 100U/ml penicillin (Sigma- Aldrich, St Louis, MO, USA) and 100 mg/ml streptomycin (Sigma-Aldrich). All the cells were incubated at 37℃ in a humidified air atmosphere containing 5% CO2. All cell lines were tested for the absence of mycoplasma contamination. Cells were used within 20 passages after thawing.

### Cell transfection and grouping assay

Cells under good state were spread on 6-well plate. When the cell density reached 50%, the siRNAs (50 nmol/L) against human TBX2 were transfected into the human lung adenocarcinoma (A549, NCI-H1299) by Lipofectamine 2000 reagents according to the instructions (Invitrogen, CA). Cells were classified into three groups: i) si-TBX2#NC group (transfected with negative control); ii) si-TBX2#1 group (transfected with si-TBX2#1); iii) si-TBX2#2 group (transfected with si-TBX2#2). After transfection, cells were incubated for 48 hours and then collected to assess the specific silencing of TBX2 expression using qRT-PCR and Western blot analysis. Also, we used this transfection cells for the later functional assay. Two small interference RNA targeted TBX2 mRNA level were designed and synthesized by RiboBio (Guangzhou, China). The target sequences showed as follows: scrambled negative siRNA was used as the control; si-TBX2 #1: GTACGAGGAGCACTGCAAA; si-TBX2#2: GCTGACGATTGCCGCTATA.

### Quantitative real‐time PCR (qRT-PCR) assay

Total RNA from cell lines were extracted using TRIzol reagent (Invitrogen, CA) and quantified with Nanodrop 2000 (Thermo, Japan). cDNA was synthesized by PrimeScript RT Master Mix (Takara, Japan). Real-time polymerase chain reaction (RT-qPCR) was performed with SYBR Green Premix Ex Taq (TaKaRa, Japan) and on an ABI Prism 7900 System (Applied Biosystems, CA). Here were all the primers used in this study: TBX2: forward (5'-TATCCTGCTGAT-GGACATTG -3'); reverse (5'-CGTGCTTG-TCAGAGATGTTG -3') and β-actin: forward (5'- TGTGGCCGAGGACTTTGATT -3'), reverse (5'-CCTGTGTG-GACTTGGGAGAG -3'). β-actin was amplified as an internal control. Relative expression differences were calculated using the 2‐ΔΔCt method.

### Cell motility and invasion assays

Migration and invasion assays were conducted in a 24-well plate with 8-mm-pore size chamber inserts (Corning, USA). For invasion assays, Matrigel (BD Biosciences, CA) was diluted to 1 mg/mL with serum free DMEM and immediately applied to the upper chamber per well. 5×10^4 and 1×10^5 cells were resuspended in serum free DMEM into the upper chamber per well for migration and invasion assays respectively. Medium supplemented with 10% FBS was added to the bottom chamber as a chemoattractant. After 18 hours of incubation at 37°C, cells that migrated or invaded through the membrane (migration) or Matrigel (invasion) were fixed with 100% methanol, stained with 0.1% crystal violet for 15 minutes and washed three times by PBS. The number of cells from the bottom was counted in 9 random fields under magnification (×100).

### Wound-healing assays

When monolayer A549 and NCI-H1299 cells grew to 100% confluent, a cell-free area was created by a sterile 200 μl pipette tip. Six microscopic fields of the migration into the gap was imaged over 0h, 24h (magnification, ×100), with an inverted microscopy equipped with a digital camera. The rate of gap closure was measured and calculated.

### Cell proliferation assay and cell-cycle analysis

Cell proliferation was assessed by Cell Counting Kit-8(CCK8) (Dojindo, Japan). Briefly, control and treated lung cancer cells (1 × 10^3/well) were seeded onto a 96-well plate for 24h. At different time points (i.e. 0h, 24h, 48h, 72 h, 96 h, 120 h), cells were incubated in a mixture of 100ul DMEM containing 10 ul CCK8 solution (Dojindo, Japan). After incubation for 2 hours, we recorded and quantified absorbance at 450 nm at each time point. For cell cycle assay, cells were harvested, fixed in 75% ethanol for 24h and stained with propidium iodide (PI) in dark for 30 minutes as provided by Cell Cycle Detection Kit (Kaiji, China) before flow cytometry analysis (BD Biosciences). Samples were analyzed on BD FACSCanto II (BD Biosciences) with data analyzed by Flow Jo (Tree Star Inc.)

### Western blot assay

Cells were collected, lysed with lysis buffer, quantified by BCA protein assay, loaded on 8 % SDS polyacrylamide gels, transferred onto polyvinylidene difluoride (PVDF) membranes (Millipore, Billerica), blocked in 5% non-fat milk and then probed with TBX2 antibody (1:500,Proteintech), E-cadherin (1:500,Santa Cruz Biotechnology), Vimentin (1:1000,Santa Cruz Biotechnology, USA), ß-catenin antibody (1:500, Sigma-Aldrich), N-cadherin (1:250, Santa Cruz Biotechnology), Slug (1:1000, Cell signaling), followed by incubation with a horse radish peroxidase-conjugated anti-rabbit or anti-mouse IgG (Sigma-Aldrich) secondary antibody. β-actin (Sigma-Aldrich, USA,1:10000) was used as the internal control. Signals were detected using Enhanced Chemiluminescence (ECL) detection system (VIAGENE, USA). The fluorescence intensity was detected with SuperSignal West Femto Maximun Sensitivity Substrate (Thermo Fisher Scientific).

### Statistical analysis

All statistical analyses were carried out using R statistical software (version3.4.2), Bioconductor packages (version 3.8), GraphPad Prism software (version 5.01) or SPSS (version 17.0.1) for Windows software. Quantitative variables were analyzed by Independent Student's tests between two groups (two-tailed). The differences in survival were calculated using the Kaplan-Meier test. All the data were representative of two or more independent experiments. Error bars in the scatter plots and the bar graphs represented the standard error of the mean (SEM). Statistical significance was defined as P < 0.05. P<0.05 (*), P<0.01 (**), P <0.001 (***).

## Results

### Preliminary screening of the original data of bone metastasis in LAC

**Figure 1:**
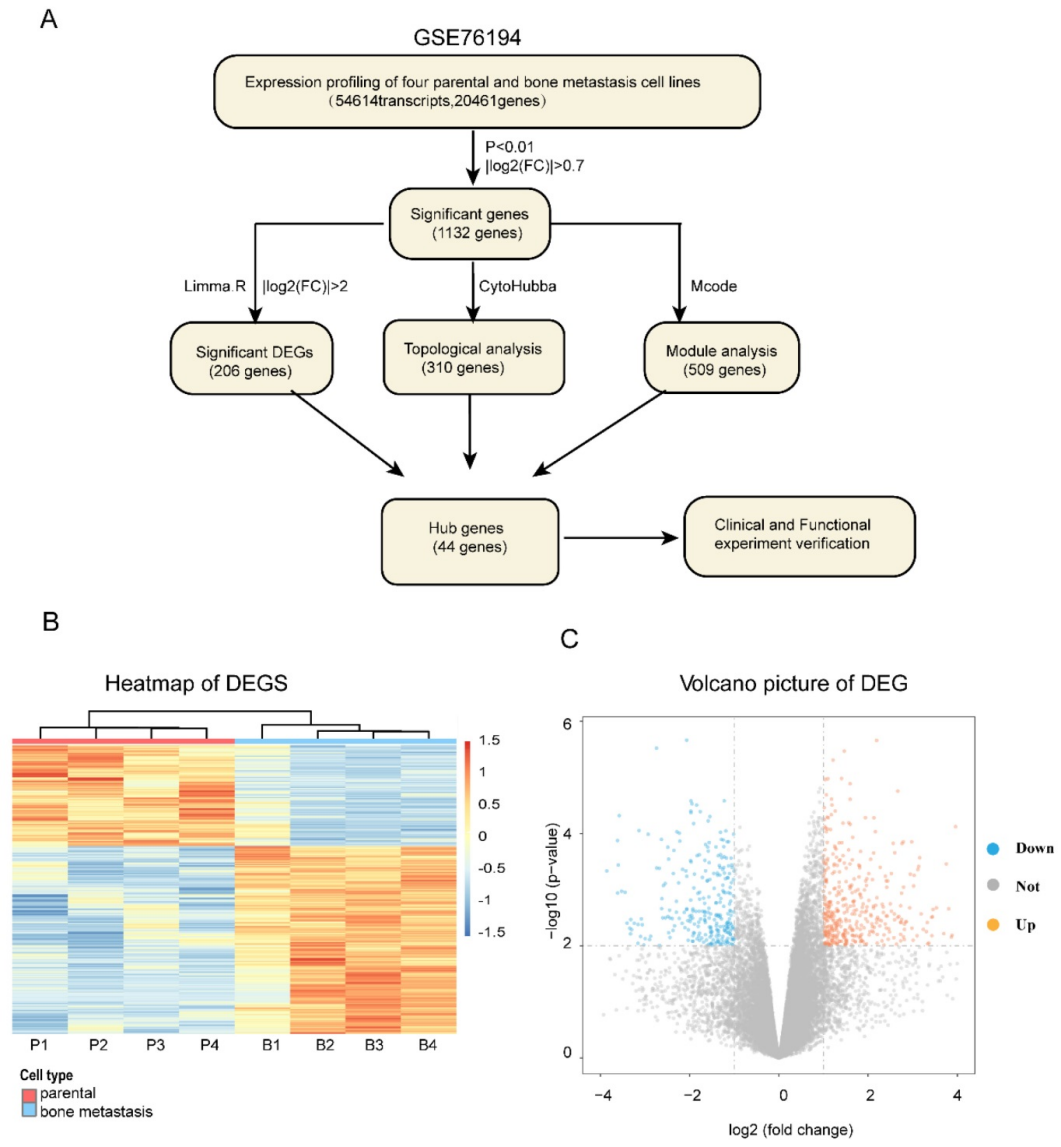
**The whole strategy and initial genetic screening for seeking hub genes. (A)** The entire workflow representing analyses and screening; Heat map **(B)** and volcano plot **(C)** visualizing the initial screening of DEGs of GSE76194 as preliminary screening. Heat map **(B)** illustrating lower (white/blue) to higher (orange/red) gene expression levels with distinct profiles across lung cancer bone metastasis. Rows are clustered by genes. Bars at the top of each column indicating the following : Red= parental; Blue =bone metastasis; For the bottom one from left to right, each column respectively representing Chinese lung adenocarcinoma cell line _parental :CPA-Yang1, CPA-Yang2, CPA-Yang3, SPC-A-1 and matched lung adenocarcinoma cell line _bone metastasis; For volcano plot **(C)**, the horizontal axis representing Log2 fold change and the vertical axis showing the reliability of -Log(P-value). The orange and blue points falling within region of interest in plot representing the differentially expressed genes, while the grey dots representing genes that are not differentially expressed, defined by| Log2(fold change) |>0.7 and P < 0.01. Blue, low expression; Orange, high expression. DEG, differentially expressed gene.

The entire flowcharts represented whole procedure involved in our current analysis and screening for hub genes related to bone metastasis in LAC, showed in Figure 1A.

We firstly obtained the gene expression profile of four pairs of LAC bone metastatic cell lines from GEO dataset (GSE76194) (Figure S1). Next, based on the differential gene analysis by Limma package in R statistical software 17, we obtained the gene expression matrix of samples. A total of 54,614 transcripts and 20,461genes were identified. As a preliminary screening step, we found 1132 significant differentially expressed genes filtered using the criteria of P<0.01 and |Log2(FC)|>0.7, of which 447 genes were upregulated and 685 genes downregulated. Here, the expression levels of differentially expressed mRNA profiling results are expressed as a heat map and volcano plot (Figure 1B, C).

### Construction of PPI network and combination of modular analysis and topology analysis

**Figure 2:**
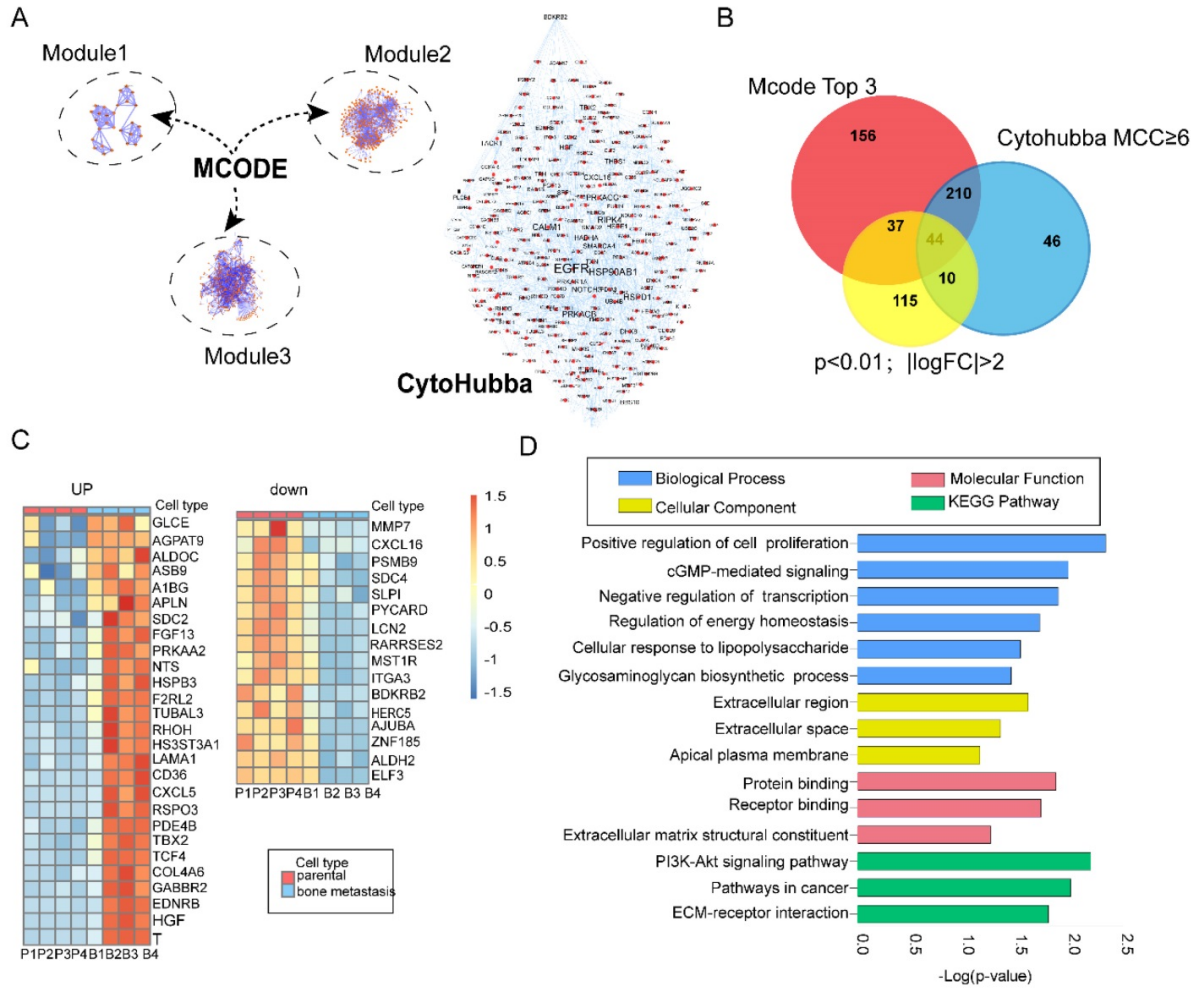
Integrated bioinformatics analysis and functional enrichment analyses. **(A)** Combination of modular analysis by MCODE and Cytohubba plugin. Left panel showing top three modules. Module 1 with MCODE score of 7.04 (nodes =28), module 2 with MCODE score of 6.517 (nodes =302) and module 3 with MCODE score of 5.325 (nodes =265). Right panel displaying different size of label related with the MCC index; **(B)** Identification of overlapping DEGs. Venn diagram representing 44 common genes among modular (447 genes), topology analysis (309 genes) and significant DEGs (Log FC>2; p<0.01,206 genes); **(C)** Overview of upregulated and downregulated overlapping genes; **(D)** GO term and KEGG pathway enrichment analyses of the overlapping DEGs. GO terms classified into three groups: biological processes (BP)、cellular components (CC), molecular function(MF). Bars represent the value of -Log(p-value).

Protein-protein interaction (PPI) is one of the best appreciated tools in understanding biological processes or molecular functions in cancer occurrence and progression 18. In the present study, we constructed a PPI network with 1415 nodes and 2018 edges based on preliminary screening 19. Next, we employed MCODE plugins in Cytoscape to find biologically essential highly connected region (subnets) and corresponding hub genes, which has been widely used in seeking hub genes in various cancer 20-22. According to the screening criteria mentioned before, we constructed three top modules in the original network (Figure 2A; Left)- module 1 had an MCODE score of 7.04 (nodes =28), module 2 had an MCODE score of 6.517 (nodes =302), and Module 3 had an MCODE score of 5.325 (nodes =265). In addition, we further carried out functional and pathway enrichment analyses of these top three highly connected regions (subnets) (Figure S2). CytoHubba is a well-known plugin which leads to new insights for inferring importance and central roles of biological networks via measuring and ranking nodes by eleven topology analysis strategies with their network features 12. Here, we evaluated the importance of each gene by Maximal Clique Centrality (MCC) and Degree (Figure 2A; Right). MCC≥6 was proposed 12. Nodes with the more forward ranking are represented. Top 10 essential nodes ranked by degree scores are shown (Figure S2).

### Identification of the overlapping genes

In total, we identified 447 and 309 genes by modular and topology analyses, respectively. Then we chose 206 common differentially genes (Log FC>2; p<0.01) for further study (Figure S3). In total, we identified 44 overlapping genes, with 28 genes upregulated and 16 genes downregulated (Figure 2C). In order to find the enrichment functional terms, we carried out GO annotation analysis and KEGG pathway enrichment analysis of DEGs using DAVID. The results showed following pathways enriched: regulation of cell proliferation, transcriptional regulation, regulation of energy homeostasis, PI3K-Akt signaling pathway and cGMP-mediated signaling (Figure 2D). P<0.05 and gene counts≥3 were considered as the cut-off criterion.

### Validation of Clinical expression and survival of Key Candidate Genes in silico

**Figure 3:**
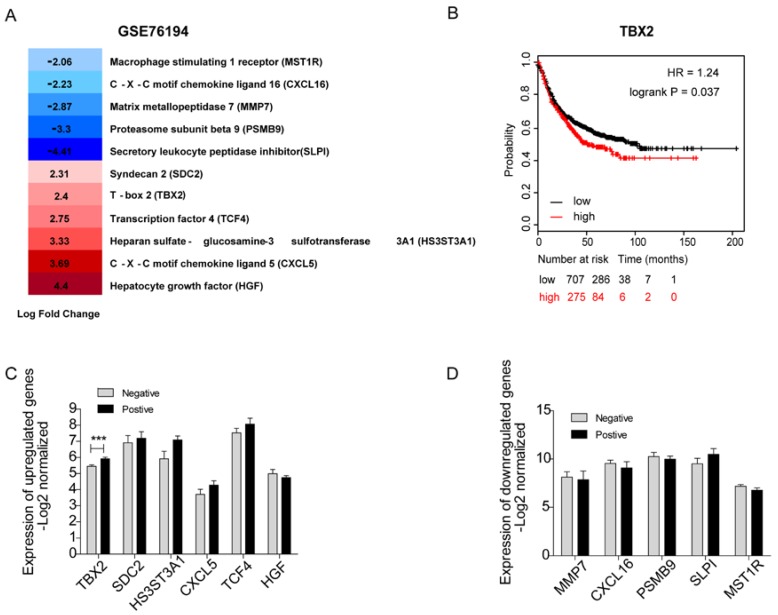
Identification of 11 candidate target genes associated with bone metastasis of LAC in silico. **(A)** Transcriptome analysis revealing differentially expressed genes of GSE76194. **(B)** Kaplan-Meier survival analysis comparing the cumulative survival rate of all patients with different TBX2 expression levels (p=0.037). Patients stratified into two groups according to median value. Affy ID utilized for analysis showed respectively as TBX2(205993_s_at). Higher expression level (red line) of those six genes was correlated with poor prognosis compared to the lower one (black line). Differences between groups evaluated using the log-rank test. HR, hazard ratio. **(C, D)** mRNA expression levels of six upregulated candidate genes **(C)** and five down regulated genes **(D)** in GSE10799. Negative meaning 9 samples in GSE10799 were found free for metastasis to bone marrow. Positive meaning 7 samples found positive for metastasis to bone marrow.

It would be ideal to obtain fresh clinical samples from paired bone metastatic patients. Unfortunately, since the existing guidelines for lung cancer generally do not recommend surgery after bone metastasis, it is difficult to obtain bone metastases in clinical patients. Therefore, we carried out a systematic and comprehensive literature search in the PubMed database of 44 underlying hub genes without any language or publication date restrictions. After scrutinizing titles and abstracts, we downloaded a full text of related publications to find out whether a relationship between those genes and bone metastasis existed. In total, we identified eleven genes that were known to play a role in bone metastasis including 5 downregulated genes-MMP7, PSMB9, SLPI, MST1R, CXCL16 and 6 upregulated genes -SDC2, CXCL5, HS3ST3A1, TCF4, TBX2, HGF (Figure 3A). We used a panel of 16 bone marrow samples from metastatic lung cancer patients (GSE10799) which included 7 samples that were positive for metastasis to bone marrow and 9 samples were negative to validate the mRNA expression level of the above genes (Figure 3C, D). Among the upregulated genes, the expression levels of SDC2, HS3ST3A1, CXCL5, TCF4 were high compared to non-metastasis samples. As to the downregulated genes (including MMP7, CXCL16, PSMB9, SLPI, MST1R), we indeed confirmed their low expression levels in patients positive for borrow marrow metastasis. However, none of them showed statistical significance. As you can easily find that in Figure 3C, TBX2 was the only candidate gene with statistically significant higher expression level in patients positive for borrow marrow metastasis, which is consistent with the gene expression data (GSE76194). First progression is defined as the length of time from the date of diagnosis or the start of treatment for a disease until the disease initially starts to get worse or metastasize 23, which is tightly associated with survival. We also evaluated first progression (FP) of 982 patients with lung cancer using Kaplan-Meier plot for TBX2. We found high expression level of TBX2 was related with the first progression and indicated poor survival (Figure 3B). These results supported that TBX2 might represent as a pivotal predictor in bone metastasis of lung adenocarcinoma.

### Silencing TBX2 suppressed migration and invasion of lung adenocarcinoma cells *in vitro*

**Figure 4:**
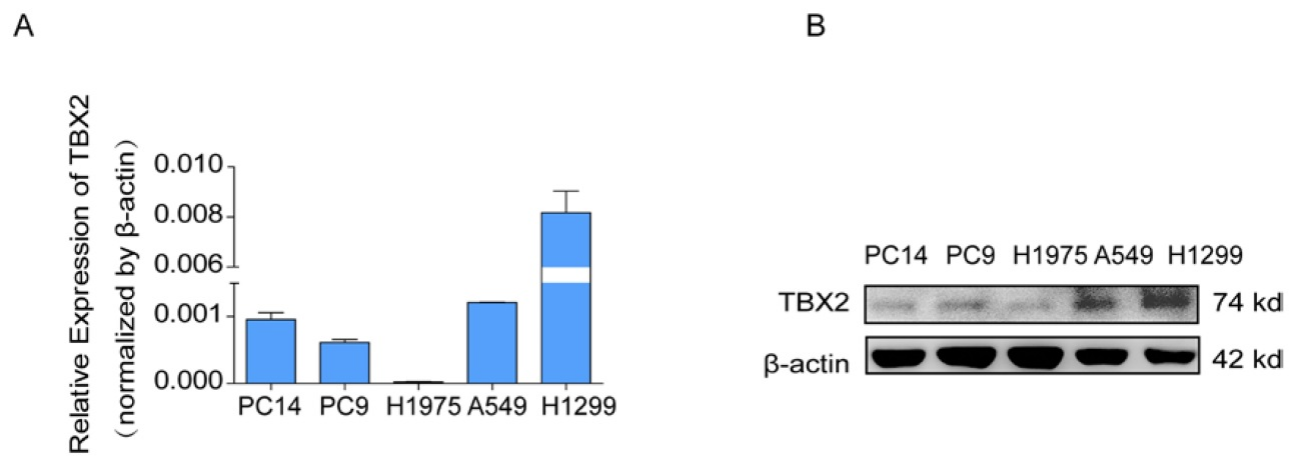
The endogenous levels of TBX2 in human LAC cell lines and reduction of aggressive cancer phenotype after knockdown of TBX2 in LAC cells *in vitro*. **(A)** TBX2 displaying frequently expression in five human LAC cell line panels (NCI-H1299、A549、NCI-H1975、PC14、PC9) in both levels of mRNA **(A)** and proteomic expression **(B )**by RT-PCR and Western blot analysis; β-actin was used as an internal control.

COPA analysis is an analytical method, termed 'Cancer Outlier Profile Analysis Cancer Outlier Profile Analysis', which was proposed by Tomlins for detecting profound changes in gene expression in cancer especially if the alterations occur in subsets of cases (Tomlins et al. Science 2005). As for its application, it has been reported that ERG and ETV1 were found as oncogenic chromosomal aberrations in prostate cancer based on this bioinformatical approach 24. Besides, SAFB was reported to be downregulated in colorectal cancer by COPA analysis, which sustained the NF-κB Pathway during the progression of colorectal cancer 25. In addition, AGTR1 was recognized as a therapeutic target in ER-positive and ERBB2-negative breast cancer cases by performing this bioinformatical approach 26. In our current study, we identified TBX2 as being markedly over-expressed in a subset of tumor samples in 20 out of 37 data sets available from Oncomine (Figure S4A) by COPA analysis. Besides, TBX2 had been reported to play a role in bone metastasis in prostate cancer. Therefore, we were interested if TBX2 was played similar functional role in bone metastasis of lung cancer. To determine whether the upregulation of TBX2 was a common event, we extended our analysis in a number of human lung cancer microarray data sets using Oncomine. Through COPA analysis, we found that TBX2 was significantly over-expressed in a subset of tumor samples in 20 out of 37 available data sets (gene rank, top 10%; fold change > 2; P < 1x 10-4) (Figure S4A). Besides, in a separate GSE29367 dataset that compares human squamous cell lung cancer line HARA with highly bone metastatic subline HARA-B4, we found it is also in concordant with our work. The expression levels of TBX2 with the presence of bone metastasis showed four times more than the parental cells, showed in Supplementary Figure S4C. Additionally, profiling from Fong's cohort (GSE5123), which reported the gene expression associated with recurrence of lung cancer, implicated an obvious and significant upregulation in the recurrent group (p<0.05), showed in Supplementary Figure S4(D). Following bioinformatics analysis, we evaluated the mRNA and protein expression levels of TBX2 in a panel of five human NSCLC cell lines (NCI- H1299、A549、NCI-H1975、PC-14、PC-9) by RT-PCR and western blot analyses (Figure 4A). We were able to detect TBX2 on both the mRNA and protein levels. In particular, the expression levels of TBX2 were higher in the A549 and NCI-H1299 cell lines than the others. To assess the functional role of TBX2 and reduce non-specific knockdown, we used two RNA interfering fragments to knock down TBX2 in A549 and H1299 cells. As illustrated in Figure 4B, we can easily find that the mRNA and protein expression levels of si-TBX2 cells have significantly suppressed compared to control in A549 and NCI-H1299 cell lines. Based on that, we evaluated the ability of migration and invasion in A549 and NCI-H1299 cell lines comparing si-TBX2#NC group and knockdown group. We found that the ability of migration and invasion were markedly suppressed (Figure 4C). Besides, in the scratch wound healing assay, we discovered that interfering with the expression of TBX2 significantly decreased the motility of NCI-H1299 and A549 cells, further suggesting that the importance of TBX2 in regulating metastasis of LAC (Figure 4D).

### Functional role of TBX2 in epithelial-mesenchymal transition

**Figure 5:**
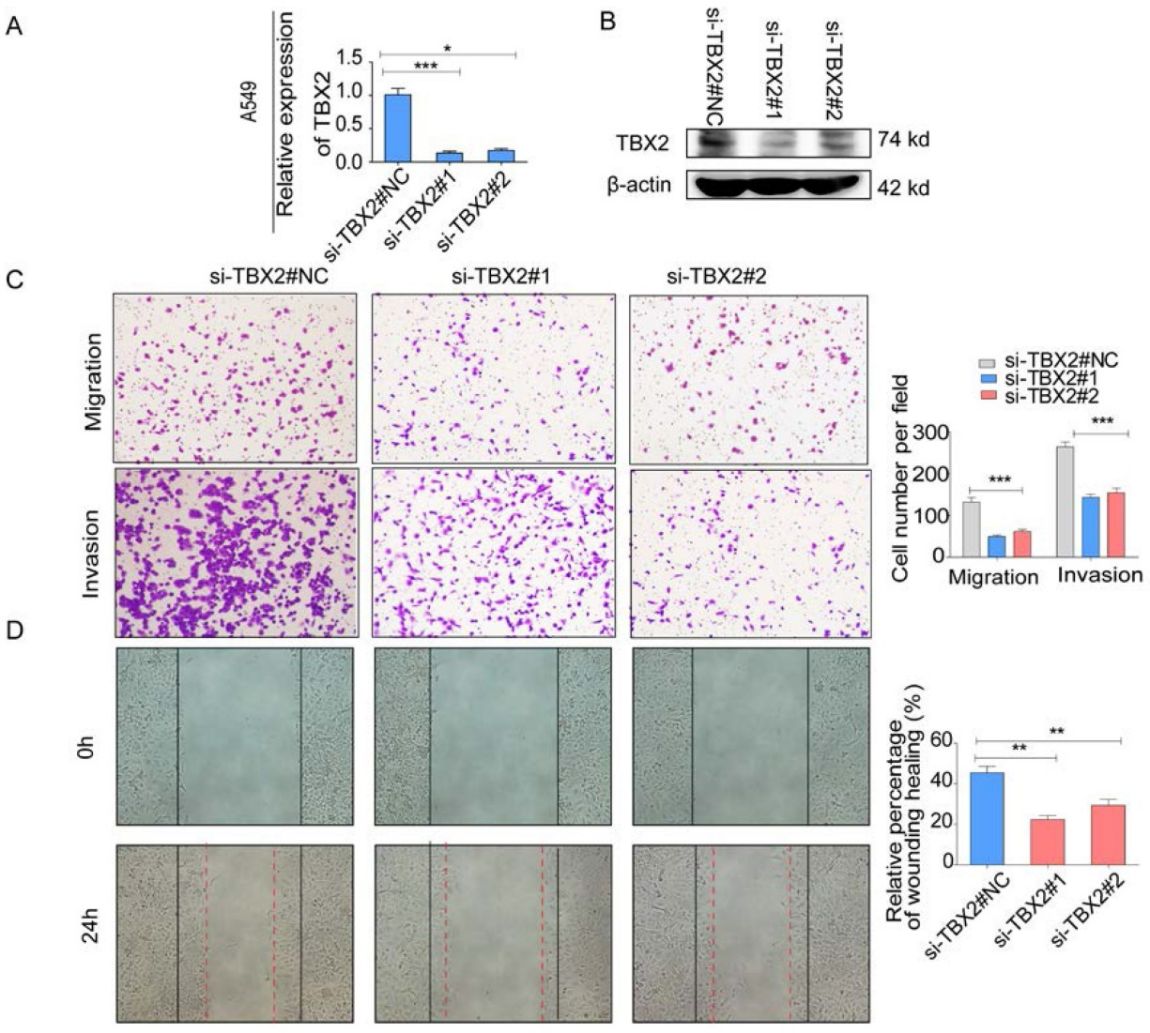
**(A,B)** Validation of TBX2 knockdown A549 at mRNA and proteomic levels after TBX2 -specific siRNAs transfection by RT-PCR and Western blot, respectively;** (C)** Reprehensive images and quantification of number of cells that migrated through a membrane or invaded through a Matrigel-coated membrane to determine the ability of migration and invasion in A549 cells after TBX2 -specific siRNAs transfection and negative control; **(D)** Representative images of initial and final wounds in A549 cells after transfection with TBX2 -specific siRNAs and negative control by wound-healing assay. Data are representative of results from three independent experiments. Error bars represent SEM *P<0.05; **P<0.01; ***p<0.001.

To gain further insight into the biologic pathways involved in bone metastasis of lung cancer, we performed GSEA analysis of the GSE76194 dataset. Gene Ontology analysis in GSEA revealed significant enrichment of gene sets related to epithelial-mesenchymal transition in bone metastasis group (Figure 5A). To validate the result of GSEA analysis, we measured the protein expression of EMT markers using western blot. Obviously, We observed markedly increased expression levels of epithelial biomarker E-cadherin(E-cad), ß-catenin(ß-cat), whereas the mesenchymal biomarkers N-cadherin(N-cad), Vimentin (Vim) as well as the EMT related transcription factor Slug were significantly lower in the experimental interference (si-TBX2) group than that in the negative control group, showed in Figure 5B, suggesting the significance of TBX2 in driving oncogenesis and metastasis.

### Silencing TBX2 suppressed cell proliferation and blocked cell cycle progression

**Figure 6:**
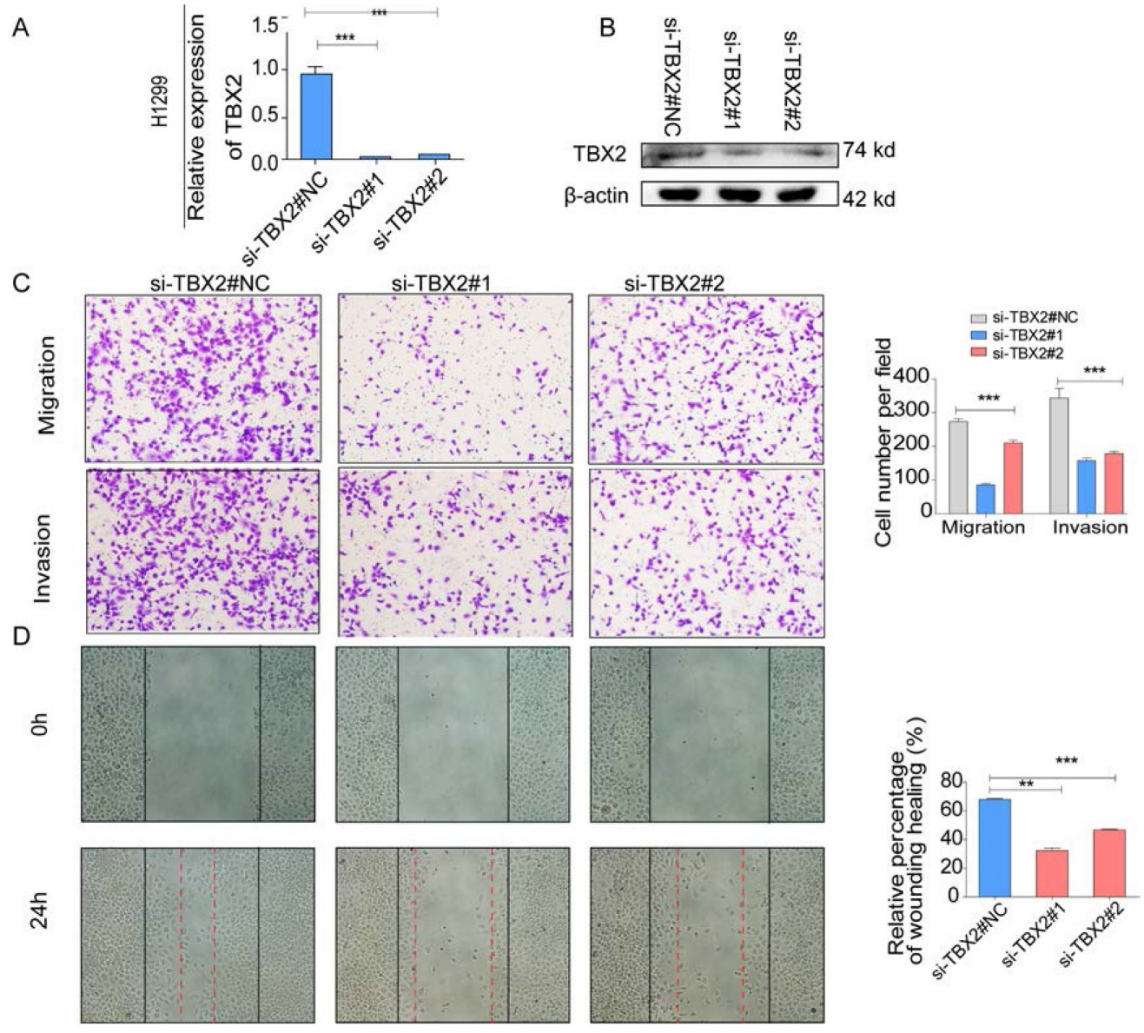
**(A,B)** Validation of TBX2 knockdown H1299 at mRNA and proteomic levels after TBX2 -specific siRNAs transfection by RT-PCR and Western blot, respectively;** (C)** Reprehensive images and quantification of number of cells that migrated through a membrane or invaded through a Matrigel-coated membrane to determine the ability of migration and invasion in H1299 cells after TBX2 -specific siRNAs transfection and negative control; **(D)** Representative images of initial and final wounds in H1299 cells after transfection with TBX2 -specific siRNAs and negative control by wound-healing assay. Data are representative of results from three independent experiments. Error bars represent SEM *P<0.05; **P<0.01; ***p<0.001.

Gene ontology and pathway enrichment revealed that the most significantly enriched categories were regulation of cell proliferation and TBX2 was in this category. In order to evaluate the effect of TBX2 on cell growth, a five-day growth curve analysis was carried out using CCK-8 assay (Figure 6A, B). We found that cell proliferation in TBX2 siRNA group was much weaker compared with the negative control group in both two cell lines, indicating TBX2 might play a key regulator in cell proliferation of lung cancer. Besides, we also indicated that TBX2 elicit a role in cell cycle. As illustrated in (Figure 6C, D), we found that silencing of TBX2 dramatically resulted in a reduced G2/M population in both A549 and NCI-H1299 cells comparing with si-NC in A549 and NCI-H1299 cells, suggesting that the reduction of cell proliferation in TBX2 knockdown cells may occur through modulating cell cycle dynamics, particularly in the G2/M phase.

## Discussion

Lung adenocarcinoma is one of the most fatal causes of cancer-related deaths worldwide, especially when metastasis occurs 27. Though modern cancer therapies are getting better, the 1-year and the 5-year survival rates among patients having lung cancer with bones metastasis are not optimistic. The rates are around 20%, 16% respectively, with a median overall survival of 9-13 months 28, 29. Skeletal related events (SRE) can seriously affect patients' quality of life and survival 30. The most captivating clinical applications of bone metastasis research is to identify “high-risk”-patients at an earlier stage. However, in the last decade biomarkers are still under intensive investigation 31. Nowadays bioinformatics programs are widely used in almost all human cancers, but different algorithms will have different results, which determine the reliability of the implementation 32. Through decades of bioinformatic analysis and studies had been performed, many of them still only provide a model for prediction. It is hard to discern between beneficial and detrimental effects of biological results or the truth just from a predictive model. In addition, though metastasis is known as one of biological the hallmarks of cancer 33, few bioinformatical analyses of metastasis and fewer on bone metastasis of lung cancer have been reported, which is inconsistent with the high mortality rate of bone metastasis of lung cancer. Therefore, with the conceptual progress of precision medicine, more research is needed to discover and develop new therapeutic targets for lung cancer with bone metastasis.

Considering all the above reasons, we performed bioinformatics-based genetic screen to identify driver genes in bone metastasis of LAC and well-designed experiments were performed for further verification. As a preliminary screening, firstly, we downloaded LAC with bone metastasis related microarray GSE76194 from GEO and analyzed DEGs. A total of 1132 significant genes with 447 genes upregulated and 685 genes downregulated. In order to visualize the data properly, we constructed a protein-protein interaction network. Through MCODE plugins, we identified the most significant three modules from the PPI network and by combining this data with the result of CytoHubba plugin, we obtained 44 overlapping genes.

To strengthen our data in a more convincing way, we searched literature for all the 44 genes and found that most of them have been reported to elicit a key role in cancer and cancer progression. For example, overexpressed A1BG has been reported in both the blood level and tumor sections of lung cancer 34; High expression of APLN is shown to significantly stimulated tumor growth and micro vessel densities; RSPO3 aberrantly expressed at high levels showed to promotes tumor aggressiveness 35; ZNF185 is investigated to inhibit growth and invasion of lung adenocarcinoma cells through inhibition of the AKT /GSK3β pathway 36. More importantly, we identified 5 downregulated genes (MMP7, PSMB9, SLPI, MST1R CXCL16) and 6 upregulated genes (SDC2, CXCL5, HS3ST3A1, TCF4, TBX2, HGF), which has been highlighted in bone metastasis. Conor C et al. has reported MMP-7 expressed at the tumor-bone interface and demonstrated a molecular mechanism between MMP-7 and osteolysis 37. PSMB9 has been investigated as molecular subgroups for therapy selection in prostate cancer, which had significantly lower mRNA level in malignant compared to nonmalignant prostate tissue and were even lower in bone metastasis tissue 38. SLPI has been reported to be deregulated with bone metastasis of lung cancer in a model that co-cultured HARA cells with calvariae 39. Alana L et.al identified that MST1R played a role in promoting osteolytic bone metastasis in breast cancer 40. SDC2, one of the cytoskeleton modulators, was reported to functioning in EMT and homing to bone 41. CXCL5 has been reported to be of great value in mediating inflammation and tumor growth in patients with bone metastasis in prostate cancer 42. CXCL16 has been described this year to play an important role in C5aR1 signaling related osteoclastogenic activity in lung cancer cells, impairing osseous colonization 43. In cell lines with high potential to multiple organs including bone, HS3ST3A1 has been reported to express highly in lung cancer tissue^44^. HGF produced by osteoblasts has been validated to induce migration of cancer cells from sinusoidal capillaries to bone marrow space and stimulates growth of cancer cells in the bone microenvironment 40. TCF4 has been validated to play a role in the regulation of breast cancer-induced bone lesions by β-catenin protein signaling 45. TBX2 has been recently underscored as a novel therapeutic biomarker in bone metastasis of prostate cancer by targeting at the TBX2-WNT signaling axis 46.

We next evaluated the expression patterns and clinical significance among them in the other independently dataset. We found that the mRNA expression levels of the above-mentioned genes related to bone metastasis were mostly consistent with the results of the GSE76194 dataset, showing a tendency to promote or inhibit bone metastasis. However, no statistically significant difference was identified due to the small sample size. TBX2 has statistically high expression levels in patients that were found positive for metastasis to bone marrow compared to patients that did not exhibit this type of metastasis in lung adenocarcinoma (GSE10799). Most importantly, Kaplan-Meier plotter revealed that higher TBX2 expression was tightly correlated with appreciably poor prognosis. In addition, gene ontology and pathway enrichment analyses suggested that the most significantly enriched category was regulation of cell proliferation in which TBX2 was found in this category. Thus, TBX2 became an obvious candidate gene of interest and we finally decided to focus on TBX2.

To our knowledge, bone metastasis of LAC is not unusual 47. T-box 2 (TBX2) is a member of the T-box family of transcription factors and involved in the morphogenesis and development of bone 48. Besides old studies have established the significant role of TBX2 in clinical cases, which showed high expression in 416 NSCLC clinical tissues and associated with highly aggressive phenotype of NSCLC 49, 50. Many reports has highlighted its role in enhancing of motility and invasiveness in various cancers and reported it to be a biomarker to predict poor prognosis in human cancers 46, 51, 52. In addition, it has been recently underscored as a novel therapeutic biomarker in bone metastasis in prostate cancer 46, but so far, the key role of TBX2 in bone metastasis of LAC had never been understood. Hence, we were interested if TBX2 was played a role in bone metastasis in LAC. Following the bioinformatics analysis, we conducted experimental verification *in vitro*. Our current study showed that TBX2 had detectable expression in LAC cell lines and in a number of human lung cancer microarray data sets. Through functional studies using siRNA transfection systems, we found silencing endogenous TBX2 expression in A549 and H1299 cell lines markedly suppressed migration and invasion, proliferation and arrest cell-cycle.

Mechanistically, here is some molecular mechanisms we hypothesized involved in TBX2 promoting bone metastasis. KEGG pathway enrichment and GSEA analysis showed the enrichment in PI3K/AKT pathway, pathway in cancer, ECM receptor iteration and epithelial-mesenchymal transition. We proved that TBX2 driving LAC oncogenesis and metastasis through epithelial mesenchymal transition (EMT) by western blot. And consistently the role of TBX2 in EMT has already been reported as well as ERK signaling pathway, triggering cell proliferation and invasion 53. Besides, there are also other pathways TBX2 involved that might promote bone metastasis. An old study implicated its role to induce tumor formation and muscle cell differentiation by repressing PTEN/PI3K/AKT pathway 54. Additionally, TBX2 has also been shown to regulate Wnt signaling pathway in canonical means and as a result of leading to metastasis and reducing bone colonizing capability 46. In addition to the above pathway, recent work has shown that TBX2 is a core regulatory circuitry component enhancing MYCN/FOXM1 reactivation of DREAM targets in neuroblastoma 55. More works need to be done to make it clear if there are other important pathways, which also represent a mechanism for the TBX2 overexpression in bone metastasis of LAC.

As TBX2 is a transcription factor, we also performed some prediction analysis on potential interacting genes that it might regulate downstream targets based on the literature and data mining. Firstly, through literature searching, we found TBX2 has been proposed to be a novel therapeutic target and as an upstream of WNT3A in metastasis for skeletal complications in patients of prostate cancer 46. It has been widely known that WNT signaling substantially impacts NSCLC tumorigenesis, prognosis, and drug resistance 56. Thus, based on this, our initial hypothesis is that TBX2 may also regulate WNT pathway during the progression of lung cancer. Consistently, we found positive and significant correlations between the mRNA expression of TBX2 and WNT3A, MMP2 in lung adenocarcinoma data of TCGA, including 515 patients of lung adenocarcinoma, illustrated in Supplementary Figure S5A, B. Additionally, high levels of MMP2 and MMP9 in the plasma of lung cancer patients have been shown to correlate with distant metastasis of lung cancer 57. Taken together, these reports and data gave our team a hint that one of the proteins of WNT pathway might be interact with TBX2. Further study has been planned and more works will be done. Besides, we have also performed some other analyses based on ENCODE, GTRD, CistromeMap datasets, which are the most complete collection of uniformly processed Chip-seq data to identify transcription factor binding sites for human and mouse. In Supplement Figure S5, we also showed the top 10 putative targets and their positive correlations with TBX2. In our future works, more functional role between TBX2 and the above predicted genes will be established.

With the development of new therapeutic strategies, this study provides the first view of screening hub genes in the pathological progression development of lung cancer with bone metastasis, by combining high-throughput data analysis and functional assays. Though we have not elaborated the specific mechanism or the microenvironmental changes when TBX2 was knocked down, further studies have been planned in our future work. Collectively, in our current study, we linked multiple bioinformatics to the biological characteristics of bone metastasis in lung cancer, yielding a more promising view towards “driver” genes responsible for bone metastasis.

## Supplementary Material

Supplementary figures and tables.

## Figures and Tables

**Figure 7:**
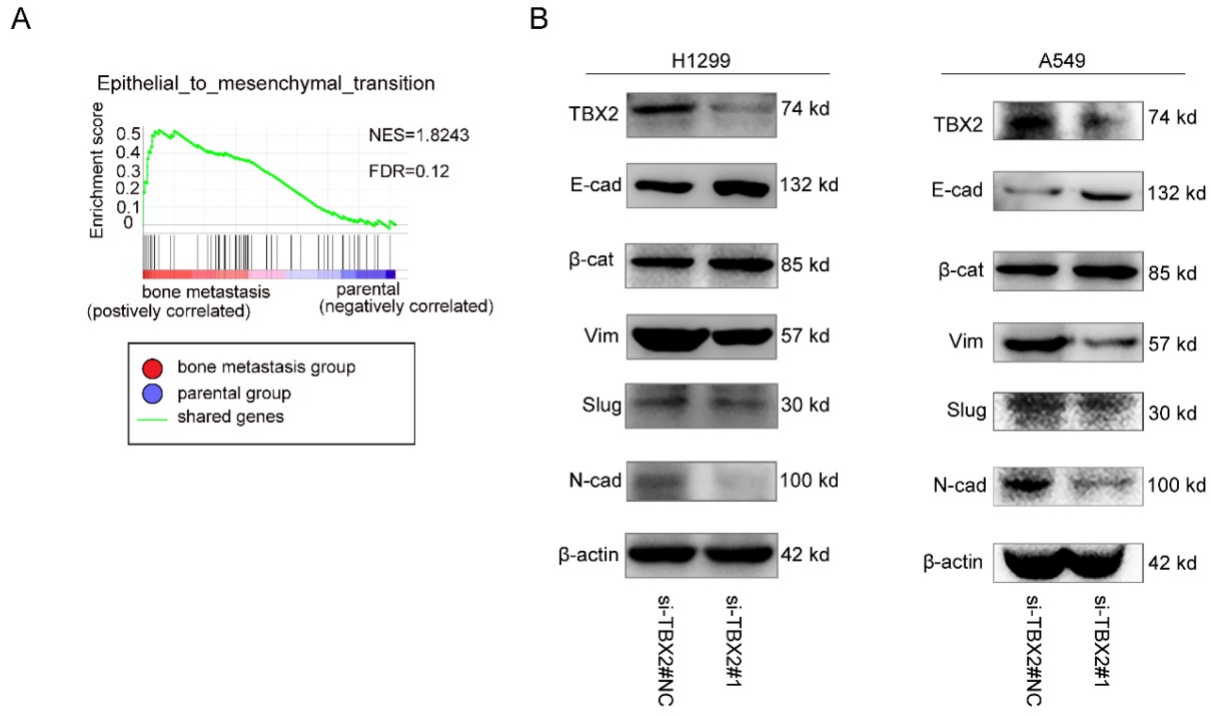
Functional role of TBX2 in epithelial-mesenchymal transition. **(A)** GO analysis in GSEA comparing bone metastasis group (red) against parental group (blue) in the GSE76194 dataset, illustrating enrichment of gene signature in epithelial-mesenchymal transition between both subgroups. FDR (False Discovery Rate) <0.25 was considered to be well-established cutoff. NES (Normalized Enrichment Score) was utilized to compare the analysis results across gene sets; **(B)** Western blot analysis of TBX2 and EMT marker expression in negative control and si-TBX2#1 group in both A549 and NCI-H1299 (E-cad = E-cadherin; ß-cat = ß-catenin; Vim = Vimentin; N-cad = N-cadherin).

**Figure 8:**
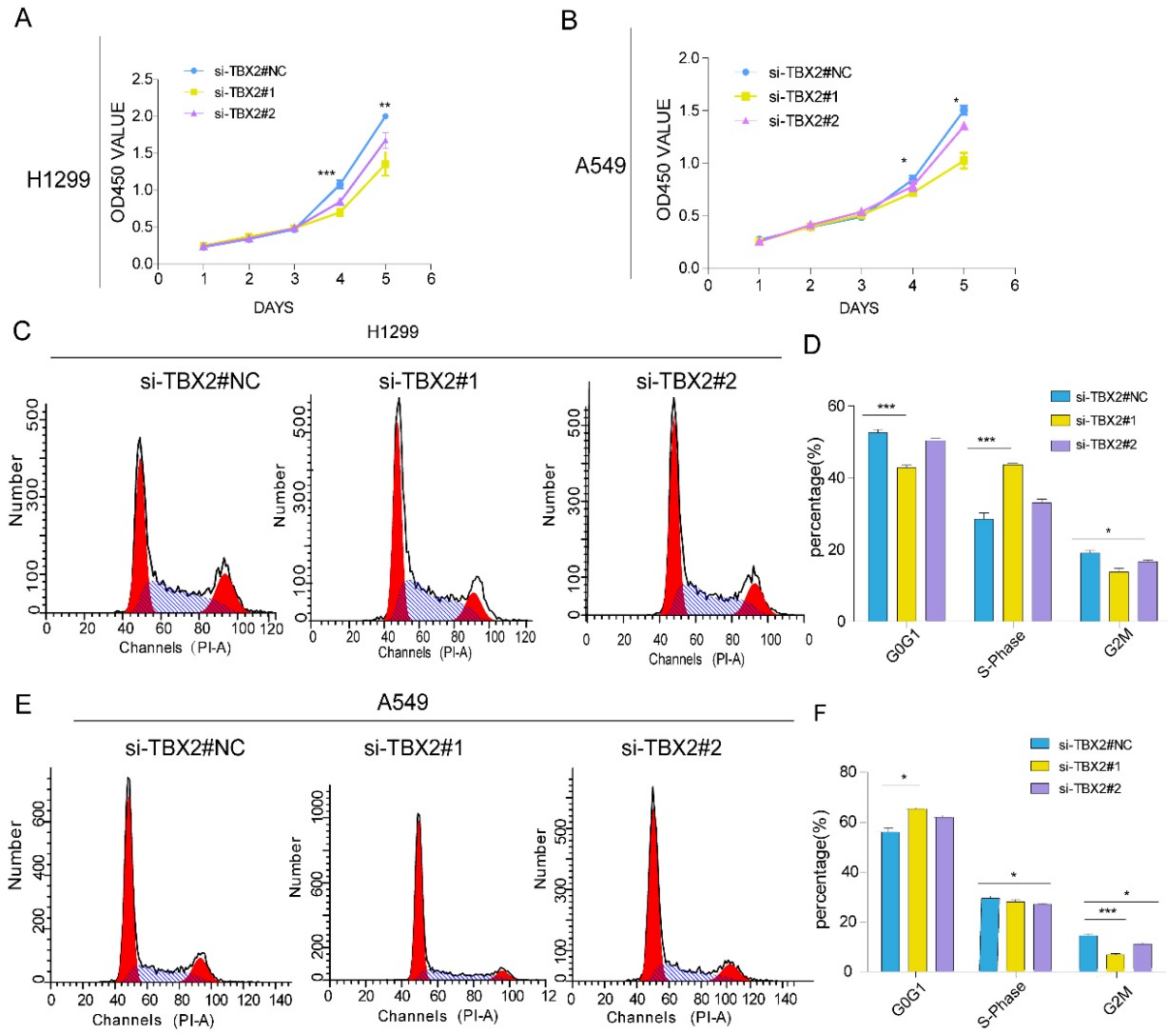
Knockdown of TBX2 abrogating cell proliferation and arresting normal cell cycle. **(A, B)** Growth curve of indicating cell lines after siRNA transfection by Cell Counting Kit-8 (CCK-8) cell proliferation assay; **(C, E)** Cell-cycle arrest after TBX2 knockdown in A549 and H1299 lung cancer cells was assessed by flow cytometry; **(D, F)** Diagrams showing the results of cell cycle assay in both indicated cell after siRNA transfection. Data and images are representative of results from three independent experiments. Error bars in the scatter and bar plots represent SEM. N=3 *P<0.05; **P<0.01; ***p<0.001.

## Abbreviations

TBX2: T-box 2
LAC): Lung adenocarcinoma
GEO: Gene Expression Omnibus
DEGs: differentially expressed genes
PPIs: Protein-protein interactions
FC: Fold change
BP: Biological Process
CC: Cellular Component
MF: Molecular Function
COPA: Cancer Outlier Profile Analysis
GO: gene ontology
KEGG: Kyoto Encyclopedia of Genes and Genomes
MCODE: Molecular Complex Detection
DMEM: Dulbecco's modified eagle medium
EMT: epithelial mesenchymal transition
E-cad: E-cadherin
ß-cat: ß-catenin
Vim: Vimentin
N-cad: N-cadherin

## References

[1] Siegel RL, Miller KD, Jemal A (2018). Cancer statistics, 2018. CA: A Cancer Journal for Clinicians.

[2] D'Antonio C, Passaro A, Gori B, Del Signore E, Migliorino MR, Ricciardi S (2014). Bone and brain metastasis in lung cancer: recent advances in therapeutic strategies. Therapeutic advances in medical oncology.

[3] Sugiura H, Yamada K, Sugiura T, Hida T, Mitsudomi T (2008). Predictors of survival in patients with bone metastasis of lung cancer. Clinical orthopaedics and related research.

[4] Gdowski AS, Ranjan A, Vishwanatha JK (2017). Current concepts in bone metastasis, contemporary therapeutic strategies and ongoing clinical trials. Science.

[5] Macedo F, Ladeira K, Pinho F, Saraiva N, Bonito N, Pinto L (2017). Bone Metastases: An Overview. Oncology reviews.

[6] Jiang P, Liu XS (2015). Big data mining yields novel insights on cancer. Nature genetics.

[7] Irizarry RA, Hobbs B, Collin F, Beazer-Barclay YD, Antonellis KJ, Scherf U (2003). Exploration, normalization, and summaries of high density oligonucleotide array probe level data. Biostatistics.

[8] Ritchie ME, Phipson B, Wu D, Hu Y, Law CW, Shi W (2015). limma powers differential expression analyses for RNA-sequencing and microarray studies. Nucleic acids research.

[9] Szklarczyk D, Franceschini A, Kuhn M, Simonovic M, Roth A, Minguez P (2011). The STRING database in 2011: functional interaction networks of proteins, globally integrated and scored. Nucleic acids research.

[10] Saito R, Smoot ME, Ono K, Ruscheinski J, Wang PL, Lotia S (2012). A travel guide to Cytoscape plugins. Nature methods.

[11] Bandettini WP, Kellman P, Mancini C, Booker OJ, Vasu S, Leung SW (2012). MultiContrast Delayed Enhancement (MCODE) improves detection of subendocardial myocardial infarction by late gadolinium enhancement cardiovascular magnetic resonance: a clinical validation study. Journal of cardiovascular magnetic resonance: official journal of the Society for Cardiovascular Magnetic Resonance.

[12] Chin CH, Chen SH, Wu HH, Ho CW, Ko MT, Lin CY (2014). cytoHubba: identifying hub objects and sub-networks from complex interactome. BMC systems biology.

[13] Huang da W, Sherman BT, Lempicki RA (2009). Systematic and integrative analysis of large gene lists using DAVID bioinformatics resources. Nature protocols.

[14] Kanehisa M, Furumichi M, Tanabe M, Sato Y, Morishima K (2017). KEGG: new perspectives on genomes, pathways, diseases and drugs. Nucleic acids research.

[15] Wrage M, Ruosaari S, Eijk PP, Kaifi JT, Hollmen J, Yekebas EF (2009). Genomic profiles associated with early micrometastasis in lung cancer: relevance of 4q deletion. Clinical cancer research: an official journal of the American Association for Cancer Research.

[16] Subramanian A, Tamayo P, Mootha VK, Mukherjee S, Ebert BL, Gillette MA (2005). Gene set enrichment analysis: a knowledge-based approach for interpreting genome-wide expression profiles. Proceedings of the National Academy of Sciences of the United States of America.

[17] Wettenhall JM, Smyth GK (2004). limmaGUI: a graphical user interface for linear modeling of microarray data. Bioinformatics.

[18] Hu Y, Zhang Y, Ren J, Wang Y (2016). Statistical Approaches for the Construction and Interpretation of Human Protein-Protein Interaction Network. BioMed Research International.

[19] Xiong Y, You W, Wang R, Peng L, Fu Z (2017). Prediction and Validation of Hub Genes Associated with Colorectal Cancer by Integrating PPI Network and Gene Expression Data. BioMed research international.

[20] Xu Z, Zhou Y, Cao Y, Dinh TL, Wan J, Zhao M (2016). Identification of candidate biomarkers and analysis of prognostic values in ovarian cancer by integrated bioinformatics analysis. Medical oncology.

[21] Li H, Liu JW, Liu S, Yuan Y, Sun LP (2017). Bioinformatics-Based Identification of Methylated-Differentially Expressed Genes and Related Pathways in Gastric Cancer. Digestive diseases and sciences.

[22] Sun C, Yuan Q, Wu D, Meng X, Wang B (2017). Identification of core genes and outcome in gastric cancer using bioinformatics analysis. Oncotarget.

[23] Bennouna J, Sastre J, Arnold D, Osterlund P, Greil R, Van Cutsem E (2013). Continuation of bevacizumab after first progression in metastatic colorectal cancer (ML18147): a randomised phase 3 trial. The Lancet Oncology.

[24] Tomlins SA, Rhodes DR, Perner S, Dhanasekaran SM, Mehra R, Sun XW (2005). Recurrent fusion of TMPRSS2 and ETS transcription factor genes in prostate cancer. Science.

[25] Jiao HL, Ye YP, Yang RW, Sun HY, Wang SY, Wang YX (2017). Downregulation of SAFB Sustains the NF-kappaB Pathway by Targeting TAK1 during the Progression of Colorectal Cancer. Clinical cancer research: an official journal of the American Association for Cancer Research.

[26] Ateeq B, Tomlins SA, Chinnaiyan AM (2009). AGTR1 as a therapeutic target in ER-positive and ERBB2-negative breast cancer cases. Cell cycle.

[27] Nichols L, Saunders R, Knollmann FD (2012). Causes of death of patients with lung cancer. Archives of pathology & laboratory medicine.

[28] Kuchuk M, Addison CL, Clemons M, Kuchuk I, Wheatley-Price P (2013). Incidence and consequences of bone metastases in lung cancer patients. Journal of bone oncology.

[29] Liu W, Wu J (2018). Lung cancer with bone metastases in the United States: an analysis from the Surveillance, Epidemiologic, and End Results database. Clinical & experimental metastasis.

[30] Brodowicz T, O'Byrne K, Manegold C (2012). Bone matters in lung cancer. Annals of oncology: official journal of the European Society for Medical Oncology.

[31] D'Oronzo S, Brown J, Coleman R (2017). The value of biomarkers in bone metastasis. European journal of cancer care.

[32] Kamali AH, Giannoulatou E, Chen TY, Charleston MA, McEwan AL, Ho JWK (2015). How to test bioinformatics software?. Biophysical reviews.

[33] Hanahan D, Weinberg RA (2011). Hallmarks of cancer: the next generation. Cell.

[34] Liu Y, Luo X, Hu H, Wang R, Sun Y, Zeng R (2012). Integrative proteomics and tissue microarray profiling indicate the association between overexpressed serum proteins and non-small cell lung cancer. PloS one.

[35] Gong X, Yi J, Carmon KS, Crumbley CA, Xiong W, Thomas A (2015). Aberrant RSPO3-LGR4 signaling in Keap1-deficient lung adenocarcinomas promotes tumor aggressiveness. Oncogene.

[36] Wang J, Huang HH, Liu FB (2016). ZNF185 inhibits growth and invasion of lung adenocarcinoma cells through inhibition of the akt/gsk3beta pathway. Journal of biological regulators and homeostatic agents.

[37] Lynch CC, Hikosaka A, Acuff HB, Martin MD, Kawai N, Singh RK (2005). MMP-7 promotes prostate cancer-induced osteolysis via the solubilization of RANKL. Cancer cell.

[38] Ylitalo EB, Thysell E, Jernberg E, Lundholm M, Crnalic S, Egevad L (2017). Subgroups of Castration-resistant Prostate Cancer Bone Metastases Defined Through an Inverse Relationship Between Androgen Receptor Activity and Immune Response. European urology.

[39] Deng X, Tannehill-Gregg SH, Nadella MV, He G, Levine A, Cao Y (2007). Parathyroid hormone-related protein and ezrin are up-regulated in human lung cancer bone metastases. Clinical & experimental metastasis.

[40] Ono K, Kamiya S, Akatsu T, Nakamura C, Li M, Amizuka N (2006). Involvement of hepatocyte growth factor in the development of bone metastasis of a mouse mammary cancer cell line, BALB/c-MC. Bone.

[41] Baniwal SK, Khalid O, Gabet Y, Shah RR, Purcell DJ, Mav D (2010). Runx2 transcriptome of prostate cancer cells: insights into invasiveness and bone metastasis. Molecular cancer.

[42] Roca H, Jones JD, Purica MC, Weidner S, Koh AJ, Kuo R (2018). Apoptosis-induced CXCL5 accelerates inflammation and growth of prostate tumor metastases in bone. The Journal of clinical investigation.

[43] Ajona D, Zandueta C, Corrales L, Moreno H, Pajares MJ, Ortiz-Espinosa S (2018). Blockade of the Complement C5a/C5aR1 Axis Impairs Lung Cancer Bone Metastasis by CXCL16-mediated Effects. American journal of respiratory and critical care medicine.

[44] Nakano T, Shimizu K, Kawashima O, Kamiyoshihara M, Kakegawa S, Sugano M (2012). Establishment of a human lung cancer cell line with high metastatic potential to multiple organs: gene expression associated with metastatic potential in human lung cancer. Oncology reports.

[45] Chen Y, Shi HY, Stock SR, Stern PH, Zhang M (2011). Regulation of breast cancer-induced bone lesions by beta-catenin protein signaling. The Journal of biological chemistry.

[46] Nandana S, Tripathi M, Duan P, Chu CY, Mishra R, Liu C (2017). Bone Metastasis of Prostate Cancer Can Be Therapeutically Targeted at the TBX2-WNT Signaling Axis. Cancer research.

[47] Reiter JG, Makohon-Moore AP, Gerold JM Minimal functional driver gene heterogeneity among untreated metastases. 2018; 361: 1033-7.

[48] Showell C, Binder O, Conlon FL (2004). T-box genes in early embryogenesis. Developmental dynamics: an official publication of the American Association of Anatomists.

[49] Zhang Z, Guo Y (2014). High TBX2 expression predicts poor prognosis in non-small cell lung cancer. Neoplasma.

[50] Hu B, Mu HP, Zhang YQ, Su CY, Song JT, Meng C (2014). Prognostic significance of TBX2 expression in non-small cell lung cancer. Journal of molecular histology.

[51] Redmond KL, Crawford NT, Farmer H, D'Costa ZC, O'Brien GJ, Buckley NE (2010). T-box 2 represses NDRG1 through an EGR1-dependent mechanism to drive the proliferation of breast cancer cells. Oncogene.

[52] Vance KW, Carreira S, Brosch G, Goding CR (2005). Tbx2 is overexpressed and plays an important role in maintaining proliferation and suppression of senescence in melanomas. Cancer research.

[53] Liu X, Miao Z, Wang Z, Zhao T, Xu Y, Song Y (2019). TBX2 overexpression promotes proliferation and invasion through epithelial-mesenchymal transition and ERK signaling pathway. Experimental and therapeutic medicine.

[54] Zhu B, Zhang M, Williams EM, Keller C, Mansoor A, Davie JK (2016). TBX2 represses PTEN in rhabdomyosarcoma and skeletal muscle. Nature communications.

[55] Decaesteker B, Denecker G (2018). TBX2 is a neuroblastoma core regulatory circuitry component enhancing MYCN/FOXM1 reactivation of DREAM targets. Nature communication.

[56] Yang S, Liu Y, Li MY, Ng CSH, Yang SL, Wang S (2017). FOXP3 promotes tumor growth and metastasis by activating Wnt/beta-catenin signaling pathway and EMT in non-small cell lung cancer. Molecular cancer.

[57] Rollin J, Regina S, Vourc'h P, Iochmann S, Blechet C, Reverdiau P (2007). Influence of MMP-2 and MMP-9 promoter polymorphisms on gene expression and clinical outcome of non-small cell lung cancer. Lung cancer.

