# Supplementary figures and images for: TBX2 Identified as a Potential Predictor of Bone Metastasis in Lung Adenocarcinoma via Integrated Bioinformatics Analyses and Verification of Functional Assay
Source: J Cancer. 2020 Jan 1;11(2):388–402. doi: 10.7150/jca.31636 (PMC6930436; doi:10.7150/jca.31636)

## Supplementary

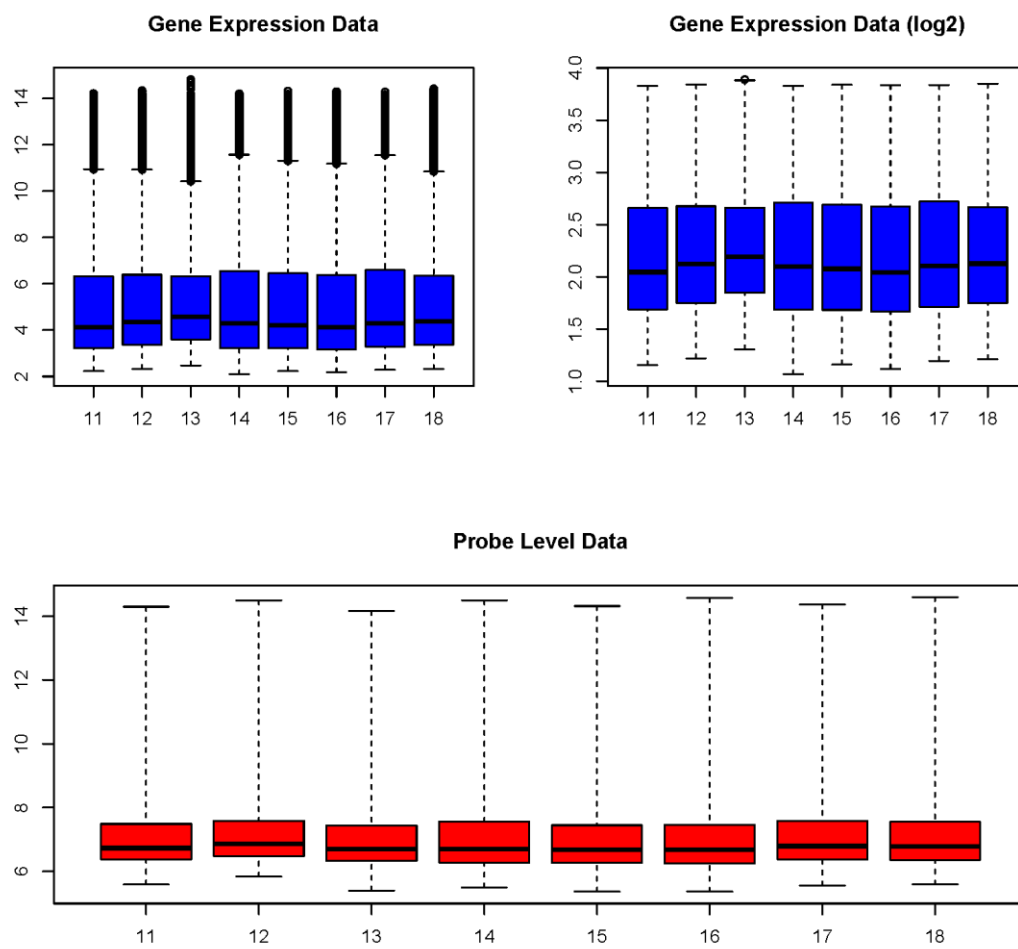

Figure S1

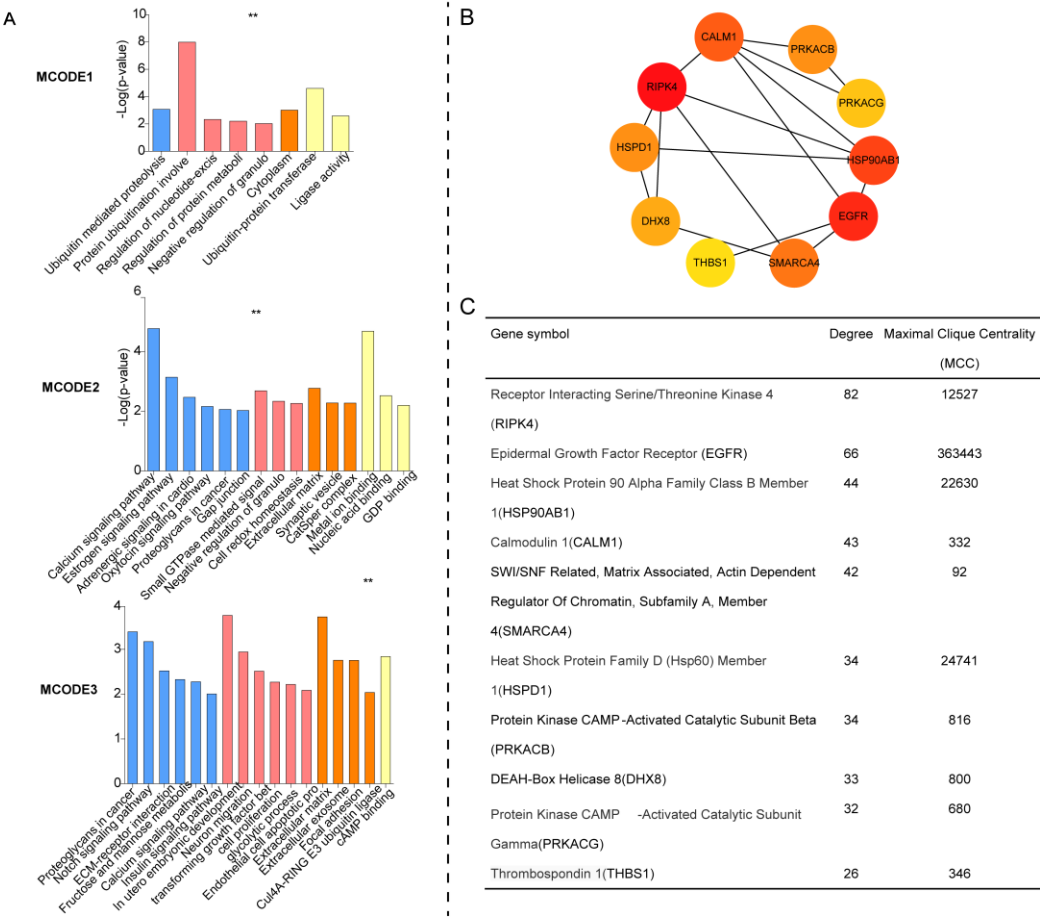

Figure S2

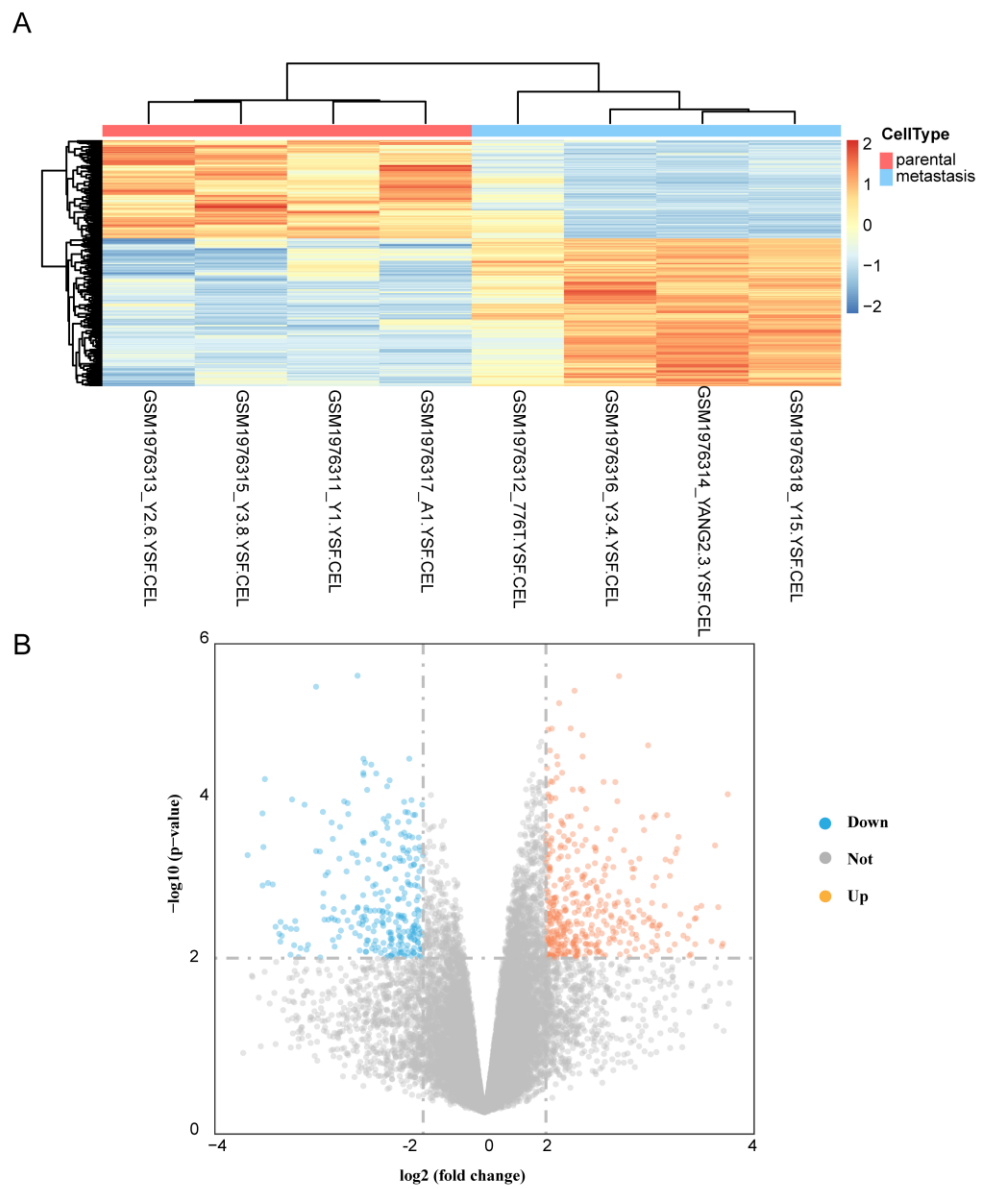

Figure S3

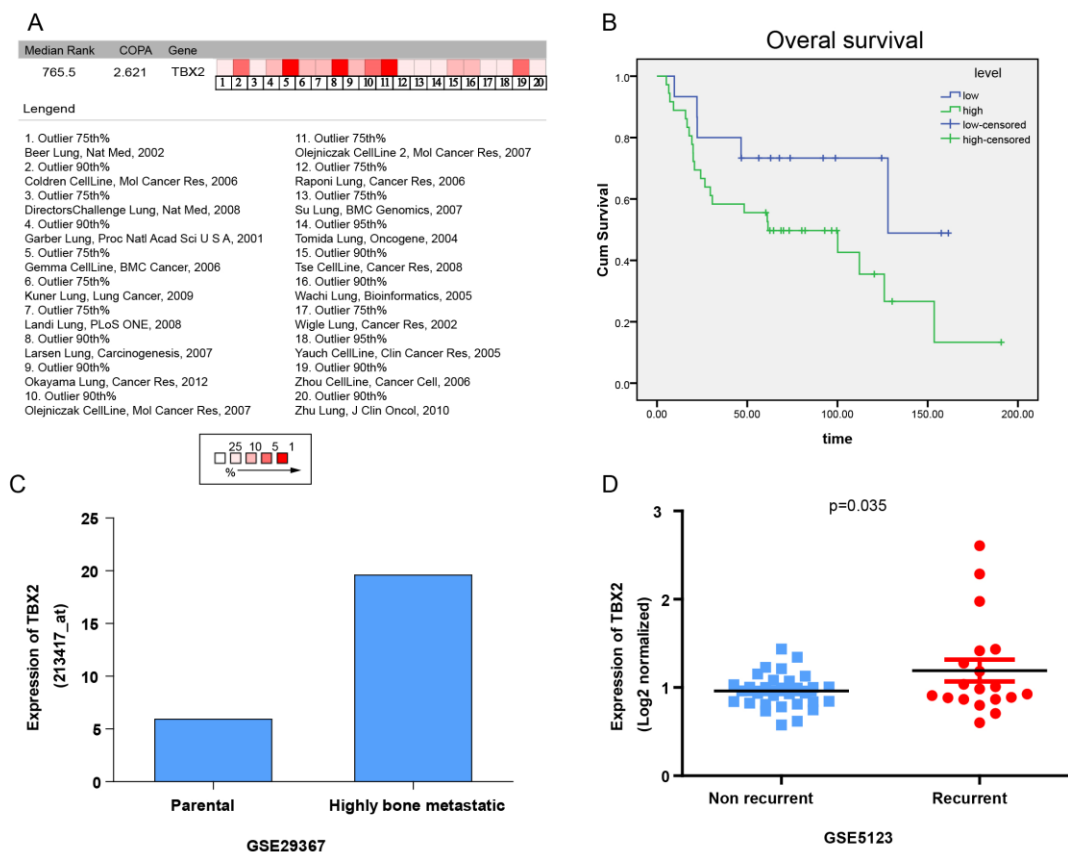

Figure S4

A

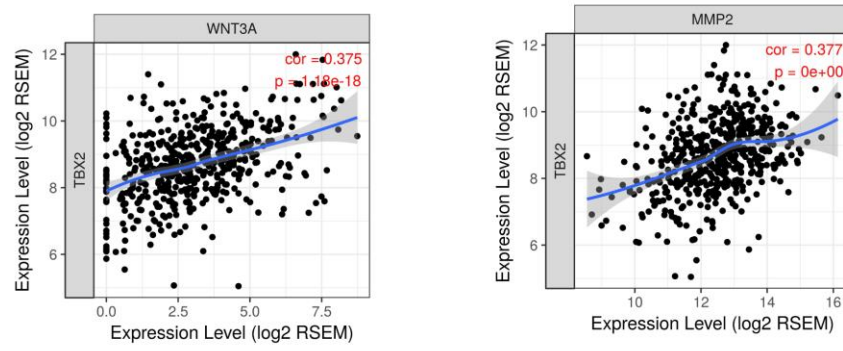

B

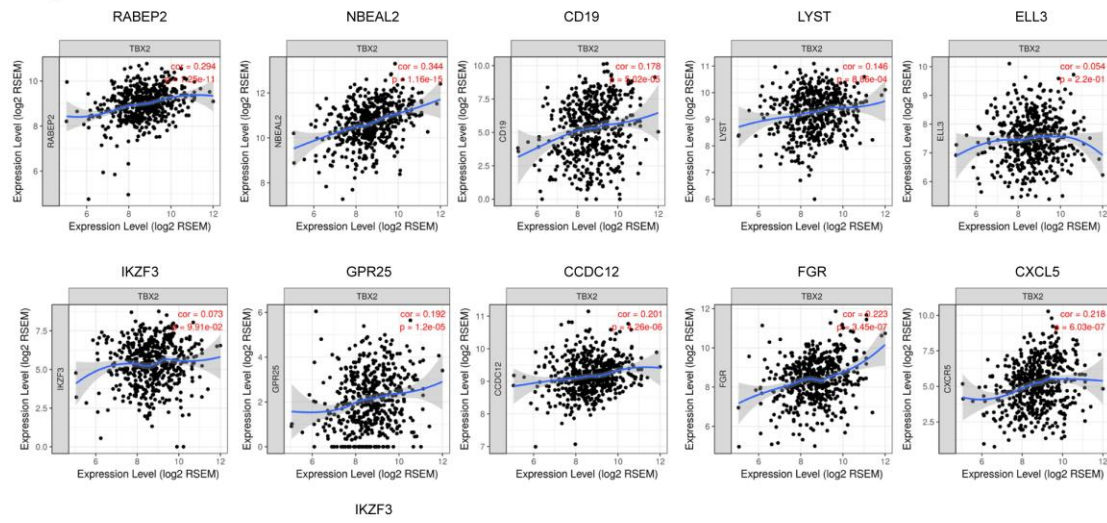

Figure S5

Supplement: Supplementary file 1 — Supplementary figures and tables. [file jcav11p0388s1.pdf]
